# Retrograde Radiological Gastrostomy Technique and Retrograde Stent Placement in a Completely Occluded Cervical Esophagus

**DOI:** 10.7759/cureus.14121

**Published:** 2021-03-26

**Authors:** Yixi Bi, Derek Edwards, Damian Mullan, Hans-Ulrich Laasch

**Affiliations:** 1 Radiology, The Christie Hospital, Manchester, GBR; 2 Interventional Radiology, The Christie NHS Foundation Trust, Manchester, GBR; 3 Radiology and Interventional Radiology, The Christie NHS Foundation Trust, Manchester, GBR

**Keywords:** esophageal stricture, esophageal squamous cell carcinoma (scc), esophageal stents, gastrostomy tube

## Abstract

Malignant obstruction of the cervical esophagus presents some anatomical and technical challenges when considering radiologic or endoscopic intervention. This case report describes the failure of antegrade access to place a gastrostomy tube and stent due to complete luminal occlusion from an esophageal tumor. The ultrasound-guided percutaneous gastric puncture was performed to achieve retrograde pneumodistension to allow radiologic gastrostomy insertion. Subsequently, the cervical esophagus was retrogradely cannulated via insertion of a guidewire from the gastrostomy site. A distal release esophageal stent was then inserted over the wire and deployed from the mouth in an antegrade manner.

However, due to the unpredictable proximal shortening of distal release stents, this stent was eventually shortened and displaced so that it no longer covered the top of the tumor stricture, and further antegrade access failed. Once more, a retrograde access approach was adopted via the gastrostomy stoma, a guidewire and catheter were passed retrogradely through the original stent and out through the mouth. A distal release stent system was then inserted in a retrograde manner via the gastrostomy stoma, effectively making it a proximal release stent which enabled more precise positioning of the stent above the tumor. Palliation was achieved until death, and beyond expected mean survival.

## Introduction

Esophageal tumors have poor five-year survival rates and pose challenges with regard to symptom control. Dysphagia, aspiration, and malnutrition are complications of esophageal cancer which can lead to a decreased survival rate and quality of life independent of the stage of the disease. Controversy remains regarding the most appropriate combination of surgery, radiotherapy, and chemotherapy in the treatment of esophageal cancer. Surgery is advised for those who are resectable and medically fit for resection [1]. Chemotherapy alone and/or radiotherapy will increase overall survival in comparison to best supportive care but can increase the frequency of grade three of four treatment‐related toxicity [2]. Dysphagia is one of the main symptoms, and in addition to being highly distressing for patients, can rapidly lead to malnutrition. Total parental nutrition (TPN), whilst a useful bridging tool for patients awaiting definitive solutions for enteral feeding, is not ideal for long-term use due to the potential for parenteral-associated liver disease, thrombophlebitis, and metabolic complications. It is well recognized that patients with esophageal cancer can struggle to maintain adequate nutrition through oral routes alone and that enteral feeding solutions can improve outcomes [3-6]. Multiple types of esophageal stents are available as palliative measures for malignant obstruction. These aim to alleviate dysphagia, improve symptom control and quality of life, and can include self-expanding metal stents (SEMS) of varying designs or biodegradable stents [7].

Cervical esophageal tumors pose particular challenges with regards to stenting because of proximity to the oropharynx which can cause pain, a foreign body sensation, as well as posing a risk for tracheal compression [8]. In cases deemed suitable for esophageal stent insertion and where there is infiltration or stenosis of the trachea, the trachea should be stented before the esophagus, in order to protect the airway [9]. There is a wide variation in esophageal stent design. Furthermore, stent lengthening and expansion varies between stent design, stent manufacturer, the degree of stenosis of the tissue to be stented, and whether the tumor is treatment naïve [10]. Most self-expanding metal stents (SEMS) use a distal release system, where the distal end of the stent opens up first upon deployment of the stent system. This can make it difficult to exactly predict the final deployed position of the proximal stent within a tight stricture. This can prove especially challenging in high/cervical esophageal tumors where the stent must be deployed proximal to the stricture, but distal to the cricopharyngeus to minimize discomfort, but still ensure adequate coverage of the tumor stenosis. Proximal release systems are designed to allow the proximal end of the stent to be deployed and expanded before the distal end is deployed. This permits a more exact proximal positioning in cases such as the cervical esophagus, where the final position of the proximal stent is important. Proximal release stents are not routinely used and are offered by fewer manufacturers [11]. We present a challenging case where conventional antegrade access for stent placement failed, requiring a non-conventional retrograde gastrostomy technique, combined with eventual retrograde stenting of the esophagus after an initial, sub-optimal, retrograde to antegrade stent placement.

## Case presentation

**Figure 1:**
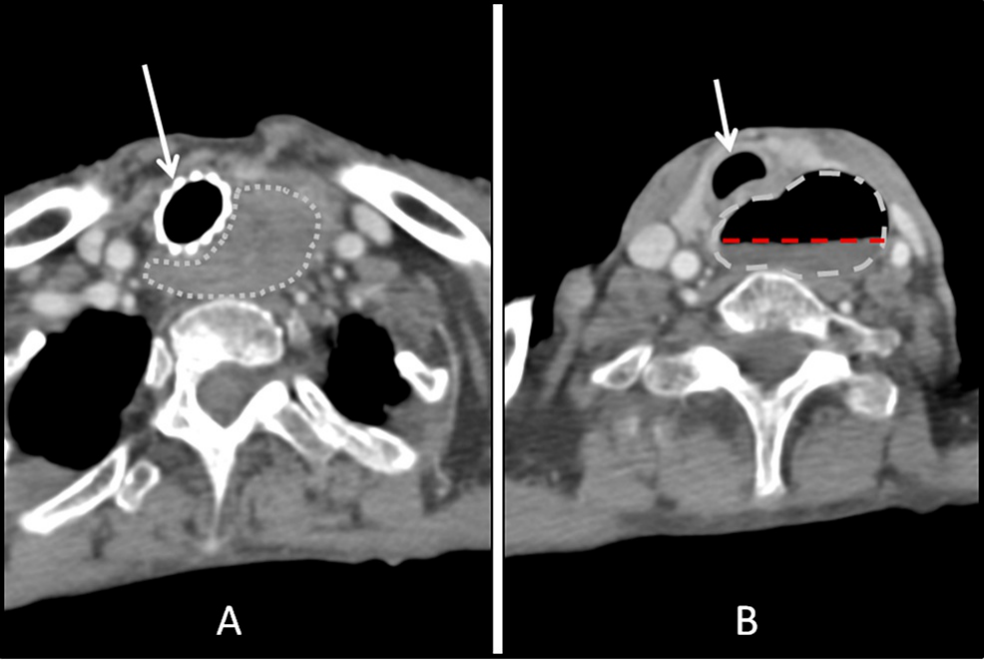
Axial CT scan of the neck and upper thorax with IV contrast performed at baseline A: Axial scan at the level of a seventh cervical vertebra showing bulky esophageal tumor mass (outlined by a gray broken line) without a clear lumen, and trachea with a tracheal stent in place (white arrow). B: Axial scan at the level of a fifth cervical vertebra showing markedly dilated esophagus (outlined by a gray broken line) and containing an internal fluid level indicative of obstruction (red broken line). The trachea is patent at this level (white arrow).

A 76-year-old woman presented with a six-week history of weight loss of 5kg and increasing dysphagia. Endoscopic assessment and biopsy diagnosed a squamous cell carcinoma of the esophagus. A CT scan showed a bulky esophageal tumor commencing at the level of the seventh cervical body, and extending over a 6cm distance into the proximal thoracic esophagus, with a very dilated esophagus proximal to the tumor (Figures 1A, 1B). Overall classification based on radiological staging was T4 N2 M0 (tracheal invasion and nodal metastases). The patient weighed 41kg at the time of diagnosis, with a body mass index (BMI) of 19.77kg/m^2^. Over the next two weeks, the patient noticed increasing dyspnoea at rest and dysphonia and was referred for a tracheal stent prior to potential gastrostomy/esophageal stent insertion. At the time of tracheal stent insertion (two weeks after diagnosis) the patient weighed 38.8kg with a BMI of 17.9kg/m^2^. Nasogastric tube (NG) insertion was attempted with endoscopic assistance, but was unsuccessful, as the lumen could not be identified through the tumor stricture. TPN was not administered pending a request for a gastrostomy tube insertion to allow enteral nutrition during possible future radical chemoradiotherapy.

**Figure 2:**
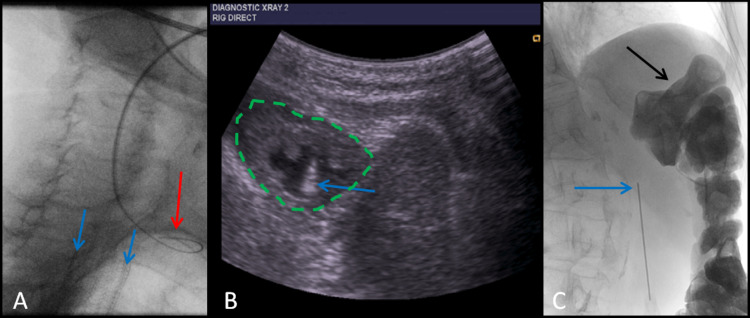
Oblique fluoroscopic images of the proximal esophagus (A), axial ultrasound of the stomach (B), and fluoroscopic image of the upper abdomen (C) A: Fluoroscopic image of failed oropharyngeal cannulation of esophagus showing coiling of the guidewire in the anterior oropharynx (red arrow). The upper borders of the tracheal stent are identified (blue arrows). B: Axial ultrasound image of the stomach (broken green line) showing successful ultrasound-guided puncture, and acoustic shadow from the Chiba needle within the stomach lumen (blue arrow). C: Fluoroscopic image of Chiba needle (blue arrow) after gastric puncture showing a paucity of air in the stomach prior to retrograde insufflation. The colon (black arrow) has been outlined by soluble contrast which was administered slowly over the preceding 48 hours.

The patient attended for a radiologically inserted gastrostomy (RIG) two weeks after tracheal stent insertion, by which time her weight had dropped to 26.2kg with a BMI of 12.1kg/m^2 ^and an ability to tolerate only small sips of water. Access for gastrostomy insertion was complicated by esophageal tumor stenosis which prevented pre-procedural passage of an NG tube (to allow barium sulfate to be given to outline the colon). Water-soluble contrast was thus administered in small sips orally 24-48 hours pre-procedure. Peri-procedural oropharyngeal cannulation of the tumor stricture to allow gastric pneumodistension was attempted with a hydrophilic guidewire and 5Fr directable catheter (Cook, Bjaeverskov, Denmark), but was also unsuccessful (Figure 2A). On fluoroscopy, no intraluminal air was identifiable in the stomach to aid with gastric localization. The stomach was localized with ultrasound and punctured with a 22G Chiba needle (Cook Medical, Bloomington, IN), as shown in Figure 2B. Figure 2C demonstrates the needle within the non-aerated stomach under fluoroscopy.

**Figure 3:**
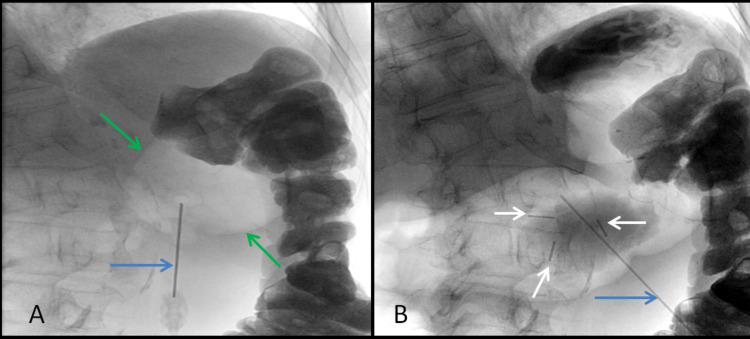
Fluoroscopic images of the upper abdomen during gastrostomy procedure A: Retrograde pneumodistension has commenced via the Chiba needle (blue arrow) and the stomach has begun to inflate, now visible as an aerated structure (between green arrows). B: Optimal pneumodistension from the Chiba needle (blue arrow) has allowed placement of three gastropexy sutures (white arrows) to secure the stomach to the anterior abdominal wall.

The stomach was then retrogradely insufflated with air (Figure 3A), and secured using three gastropexy sutures (Vygon, Cirencester, UK) (Figure 3B). A 12 French balloon gastrostomy tube (Vygon, Cirencester, UK) was placed. The gastrostomy allowed enteral feeding to resume, and the patient gained 10kg of weight over the next three weeks.

Although the patient had no significant co-morbidities, it was concluded that they would not be fit enough for radical chemoradiotherapy given a declining performance status. The patient declined palliative radiotherapy, opting for best supportive care, with a referral for esophageal stent placement to allow oral nutrition and improve remaining quality of life.

**Figure 4:**
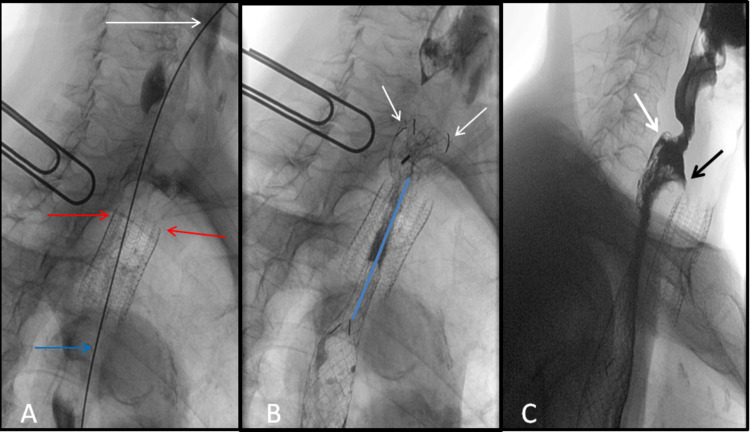
Sequential fluoroscopic oblique views of the upper thoracic esophagus during esophageal stent insertion A: A stiff guidewire (blue arrow) has been passed retrogradely from the gastrostomy stoma, through the middle and upper thoracic esophagus, through the cervical esophagus (white arrow), and exiting through the mouth to allow subsequent antegrade esophageal stent insertion. The tracheal stent (red arrows) is superimposed upon the course of the esophagus on this two-dimensional fluoroscopic image.  A paperclip has been placed on the skin of the patient at the level of C7 to further aid localization of anatomical landmarks. B: Image of esophageal stent immediately post-deployment showing satisfactory coverage of the tumor stricture (blue line) and good expansion of the upper stent (white arrows) indicating adequate coverage of the proximal tumor. C: Water-soluble contrast swallow study one day post stent insertion showing a patent stent outlined by contrast, but with marked eccentric shortening of the anterior proximal stent (black arrow) and mild shortening of the posterior proximal stent (white arrow).

Three weeks after gastrostomy insertion, the patient attended for stent insertion. Antegrade access failed once more. A wire was then inserted retrogradely through the gastrostomy stoma site, and retrograde cannulation of the tumor stenosis was performed (Figure 4A) with the passage of the wire out through the mouth. A distal release 18 x 110mm covered esophageal stent (EGIS, S&G Biotech, Yongin, Korea) was then deployed in an antegrade manner from the mouth with the proximal end of the stent covering the proximal tumor, adjacent to the upper border of C7 (Figure 4B). The procedure was uncomplicated and the patient was started on small volume oral fluids to build up to a semi-solid diet as tolerated. A contrast swallow the next day showed the stent had expanded, but shortened. It remained at the top of C7, just covering the most proximal part of the stricture. There was free flow of iopamidol contrast through the stent (Figure 4C) and a semisolid diet was commenced. One-week post-procedure the patient had gained 2kg to 38kg with a BMI of 17.6kg/m^2^.

**Figure 5:**
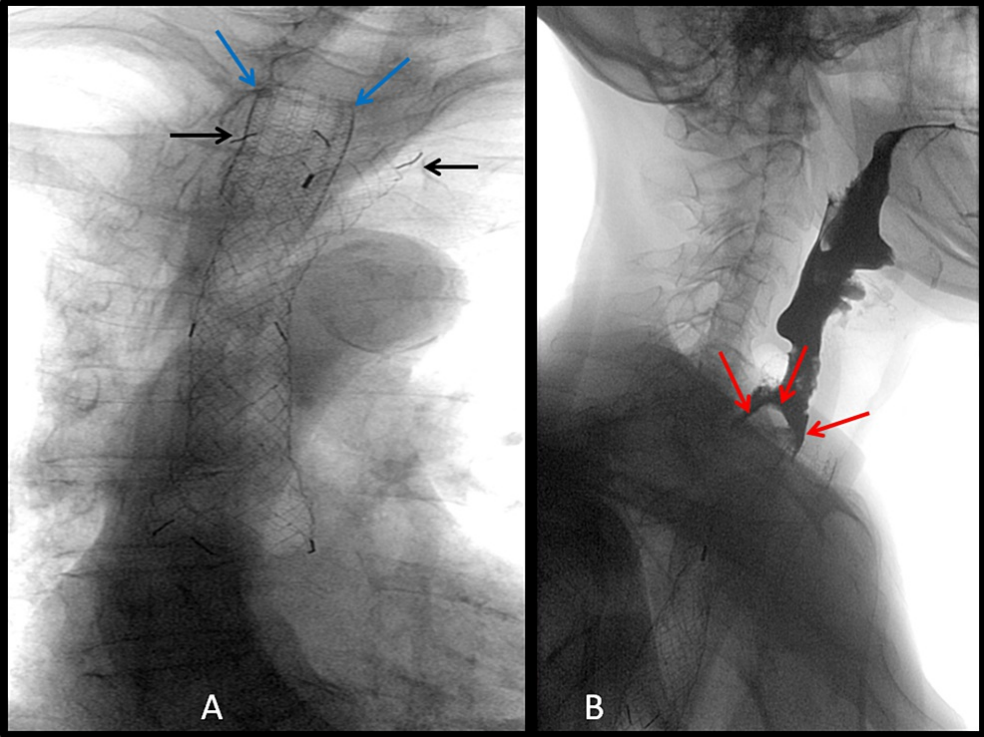
Fluoroscopic images from water-soluble contrast swallow were performed due to new dysphagia following previous esophageal stent placement A: An initial image without water-soluble contrast shows the proximal markers of the esophageal stent to have expanded fully (black arrows), but has now shortened, and migrated to below the level of the tracheal stent (blue arrows). B: A contrast swallow examination shows a failure of contrast to outline the esophageal stent with a bulbous filling defect proximal to the esophageal stent (red arrows) indicating that the stent no longer covers the tumor stricture.

Twenty days after stent insertion, the patient complained of new dysphagia, only managing small sips of water. Fluoroscopy showed that the esophageal stent had expanded and shortened, migrating inferior to the tracheal stent, and no longer covering the proximal tumor stenosis (Figure 5A). A water-soluble contrast swallow showed no passage of oral contrast through the stent (Figure 5B).

**Figure 6:**
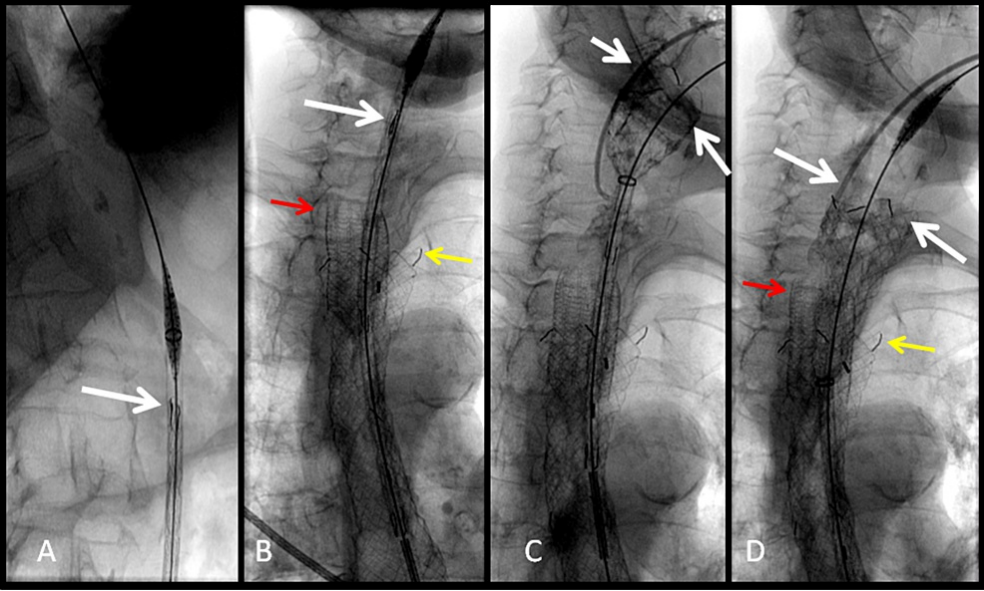
Fluoroscopic images of retrograde esophageal stent insertion A: The stent (white arrow) is advanced in a retrograde fashion from the gastrostomy access. B: The stent (white arrow) is passed in a retrograde fashion coaxially through, and above the level of the existing esophageal stent (yellow arrow), and above the level of the tracheal stent (red arrow). C: The distal release stent is retrogradely deployed above the tumor stricture with a good expansion of the stent above the tumor (white arrows). D: The esophageal stent is fully deployed with full expansion of the stent above the tumor stricture (white arrows) and above the shortened existing esophageal stent (yellow arrow), and above the level of the tracheal stent (red arrow).

A further attempt at stent insertion was performed. There was again complete tumor stenosis which prevented antegrade guidewire and catheter passage from the mouth. The gastrostomy tube was removed and retrograde access of the esophagus was again obtained from the gastrostomy stoma (Figure 6A). A guidewire and catheter were threaded through the indwelling esophageal stent and pulled out through the mouth. A distal release stent system was advanced through the gastrostomy and a 20 x 90mm nitinol-covered EGIS stent (S&G Biotech, Yongin, Korea) was delivered in a retrograde manner (Figure 6B) effectively making it a proximal release stent system (Figure 6C). This technique allowed a more precise placement, covering the proximal tumor stenosis more adequately than the original stent (Figure 6D).

After the second esophageal stent, the patient was able to resume a soft diet orally and receive nutritional supplementation via gastrostomy feeds. Two months after her second esophageal stent her weight had further increased to 39kg with a BMI of 18.0kg/m^2^, and the patient was successfully palliated with best supportive care until death, surviving for 10 months after the initial diagnosis, and eight months after the second esophageal stent placement.

## Discussion

Approximately 90% of patients with esophageal cancer will experience dysphagia, often making it impossible to provide adequate nutrition orally [4]. Esophageal stents are one of the most commonly used methods for palliative control of dysphagia. An antegrade trans-oral approach is the accepted method for stenting tumor stenosis.

Insertion of stents in the cervical esophagus is challenging anatomically and technically. Stenting the cervical esophagus can threaten the airway due to tracheal compression from the expanded esophageal stent. Therefore, it is commonly accepted that a tracheal stent should be considered prior to stenting the esophagus [8,9,12].

In cases of complete dysphagia, parenteral feeding can be used for nutrition, but it is recognized that the enteral route is preferable due to improved immune response, butter gut function and reduced length of hospital stay, and decreased complication profile [3-5]. Percutaneous endoscopic gastrostomies (PEG) are the most commonly used form of gastrostomy worldwide. This patient was not suitable for a PEG insertion due to an inability to identify the lumen, and an inability to pass the scope beyond the tumor. Tumor seeding is an uncommon but recognized concern when traversing a tumor to perform PEG insertion [13]. Alternatively, RIGs require passage of a small-bore NG tube for gastric insufflation and therefore have a marginally higher technical success rate compared to PEGs [14]. RIG insertion is reported as safe, with a low complication profile [15].

The conventional method for creating gastric pneumodistension is via a nasogastric tube, which allows air to be insufflated anterogradely. This enables visualization of the stomach on fluoroscopy and reliable percutaneous puncture of the stomach for subsequent RIG tube insertion. Ultrasound-guided puncture of the stomach is not conventionally used due to gastric air causing degradation of ultrasound images, preventing accurate localization of the stomach. However, some studies have demonstrated that ultrasound-guided percutaneous puncture of the collapsed stomach is feasible [16]. In this case, the patient’s complete dysphagia meant that no air was visible fluoroscopically in the stomach. Conversely, this enabled a more reliable ultrasound localization of the stomach, enabling direct puncture and needle insufflation.

Retrograde pneumodistension of the stomach enabled a RIG to be inserted which provided a valuable feeding solution for the patient. Furthermore, the mature gastrostomy stoma was later used to enable retrograde guidewire access and catheter insertion which was used for insertion of the first esophageal stent which was inserted in an antegrade manner. Stenting of the cervical esophagus can be technically challenging, and a placement that is too close to the cricopharyngeus muscle, or proximal migration can lead to a foreign body sensation within the oropharynx [17]. Antegrade access can be difficult, and some case reports describe a 'rendezvous' technique whereby retrograde access is converted to antegrade stent placement [18]. Accurate antegrade placement of a conventional distal release stent can be challenging due to unpredictable stent expansion, as was the case for the initial stent in this case. Such patients may benefit from the use of a proximal release stent for more accurate positioning during deployment. In this case, the second stent insertion was performed with a conventional distal release stent but inserted in a retrograde fashion, ultimately converting it into a ‘pseudo’ proximal release stent, which protected the oropharynx by enabling a more precise placement.

## Conclusions

In cases with limited or no antegrade esophageal access, retrograde pneumodistension can enable the insertion of a radiologic gastrostomy tube. Ultrasound-guided direct puncture is not routine but is possible. In cases where per-oral cannulation of a tumor stricture is impossible, the gastrostomy stoma can then be used for retrograde guidewire insertion, luminal cannulation, and retrograde stent insertion. Due to unpredictable shortening/expansion of stents within tumor strictures, proximal release stents may be of more value when stenting the upper thoracic/cervical esophagus. Stenting the tumor retrogradely from the gastrostomy stoma access allows a conventional stent to be used as a proximal release stent to achieve precision stenting. To the authors' knowledge, this is the first case in the literature with a combined retrograde pneumodistension approach for gastrostomy and subsequent retrograde stent insertion.
